# Longitudinal dataset of journal data sharing policies across 22 disciplines for 2014 2019 and 2023

**DOI:** 10.1038/s41597-025-06434-2

**Published:** 2025-12-13

**Authors:** Ui Ikeuchi

**Affiliations:** Department of English Language and Literature, Faculty of Language and Literature, Bunkyo University, Koshigaya, Japan

**Keywords:** Research data, Publishing, Policy, Research management

## Abstract

This dataset documents journal data sharing policies across 22 disciplines for 2014 2019 and 2023. A total of 220 high-impact journals were surveyed, representing the top ten journals (by Impact Factor) from each discipline listed in the Essential Science Indicators. For each journal, policies concerning two types of data sharing were reviewed: repository-based data sharing and supplementary materials. Policy requirements were classified into four categories based on their strength: require, recommend, accept, or no policy. Data were collected at three time points through systematic reviews of journal websites and submission guidelines. The dataset includes journal metadata—such as publishers, ISSN, and Impact Factor—along with detailed policy descriptions and classifications. This longitudinal dataset provides evidence of changes in data sharing requirements over time and enables comparative studies of journal policies. These data may be useful for research on open science practices, the development of science policy, and the evolution of scholarly publishing standards.

## Background & Summary

### Rationale for dataset creation

Sharing research data enhances the visibility^1–3^ and transparency^4–6^ of scholarly work. Furthermore, making research data available in accordance with the FAIR principles increases the potential for reuse and contributes to the advancement of open science^7,8^. In parallel, data journals^9^ and disciplinary data repositories^10^ have evolved to promote formal data publication, citation, and long-term preservation of data, recognising datasets as a “first-class research output” distinct from traditional scholarly publications.

Journal data sharing policies are recognised as a key driver for researchers to disclose their data^11,12^. Studies show that journals with stronger data sharing requirements tend to exhibit higher levels of data availability^13,14^. Against this background, various guidelines have been published^15–17^, including publisher-level policies^18–20^ and recommendations from the International Committee of Medical Journal Editors (ICMJE)^21^. Nevertheless, the strength of data sharing requirements differs among journals^22,23^ and varies across disciplines^24–26^. These policies have evolved over time^27^. To address this issue, a cross-disciplinary and longitudinal survey of journal data sharing policies was conducted.

### Potential for reuse

Stodden *et al*. conducted surveys in 2011 and 2012 on data sharing and code sharing policies in computational science journals, made these data publicly available, and reported on longitudinal changes^28^. Similarly, the dataset presented here enables comparative research on the evolution of journal data policies over time and across disciplines.

This dataset comprises data sharing policies and journal metadata for the years 2014, 2019, and 2023. The target set consisted of the top ten journals by Impact Factor in each of the 22 subject categories defined by the Essential Science Indicators, resulting in a total of 220 journals. For each journal, two types of policies were reviewed: (i) data sharing policies requiring data deposition in repositories and (ii) supplementary materials policies allowing data to be provided on the journal platform. Journal metadata were also collected.

In addition to the core review of journal policies, supplementary investigations were undertaken. For the 2014 dataset, designated or recommended repositories were recorded, as depositing data in recognised subject or public repositories is considered an essential element of effective data sharing^6^. For the 2019 and 2023 datasets, policies relating to data citation were also examined, as providing formal credit through data citation is regarded as a key incentive for researchers to share their data^29^. These supplementary investigations enhance the reuse potential of the dataset by enabling users to explore not only the presence of data sharing requirements but also how journals encouraged the use of trusted repositories and the acknowledgement of shared data through citation. The shift from recording repository designations (2014) to examining data citation policies (2019–2023) reflects the evolving emphasis on citation-based incentives following the publication of the FORCE11 Joint Declaration of Data Citation Principles (JDDCP)^30^ in 2014.

The author previously used the 2014 dataset to examine the characteristics of journals requiring data sharing^31^ and to compare these requirements with the actual data sharing practices of Japanese researchers, identifying disciplines where data sharing lagged behind requirements and where policy or institutional support might be needed^32^. A comparison of the 2014 and 2019 datasets was used to assess longitudinal changes and determine whether data citation statements were included^33^. Furthermore, a difference-in-differences analysis across the three survey years suggests that journals which introduced data sharing requirements tend to show an increase in their Impact Factor values^34^.

Of the datasets released here, the 2014 version was previously published^35^; however, additional information, such as policy descriptions, has been incorporated. The classification criteria were revised based on three rounds of surveys. The 2019 and 2023 datasets are newly published. These data enable comparisons of changes in the proportion of journals requiring data sharing and allow the examination of the influence of data sharing policies over time.

## Methods

### Journal selection (2014)

The sampling design was guided by prior evidence and a preliminary cross-disciplinary review conducted by the author. Previous studies have shown that journals with higher Impact Factors are more likely to publish explicit data sharing policies^14,28,36^. A preliminary review of 180 journals^37^ further indicated that lower-impact journals rarely published explicit data sharing policies, making reliable cross-field comparison difficult.

The same review also found that review, commentary, short communication, and news journals seldom contained original datasets. Therefore, the present dataset intentionally targets *high-impact, original-research-oriented journals* to ensure consistency and comparability across disciplines.

Journal subject classification was based on Essential Science Indicators (ESI), a bibliometric database produced by Thomson Reuters (now Clarivate). The journals in the ESI Journal List^38^ are uniquely assigned to one of 22 subject areas, covering the natural sciences, life and medical sciences, and social sciences. The September 2013 version of the Journal List included 11,872 titles.

First, 27 titles without ISSNs and not listed in the 2012 edition of the Journal Citation Reports (JCR)^39^ were excluded, leaving 11,845 journals. Impact Factor values were obtained from JCR (2012), and journals were ranked within each subject area.

Second, journals primarily publishing original research articles were retained. Review, news, short communication, and commentary journals were excluded, except in cases where journals with “review” in the title published mostly original articles.

Third, the top ten journals in each of the 22 ESI subject areas were selected, resulting in a total of 220 journals.

Metadata were collected from Ulrichsweb^40^ (publisher, country, year of first publication), from JCR and journal websites (ISSN, URL), and from Google Scholar Metrics^41^ (2013). Only aggregated and non-identifiable metadata, such as journal titles, ISSNs, publisher names, and Impact Factors, were extracted for analysis. These elements are publicly visible on the corresponding journal pages and were used in compliance with Clarivate’s Terms and Conditions (version 3.2, March 2024)^42^, which permit fair academic use but prohibit redistribution of proprietary database content.

Additional bibliometric indicators (h5-index) were obtained from Google Scholar Metrics (GSM), which publicly lists top-ranked journals by citation metrics. Only aggregated, factual information—journal titles and citation indicators displayed on the GSM website—was used. These data are publicly available and were used in compliance with Google’s Terms of Service (effective May 22, 2024)^43^, which permit the use of publicly accessible content for non-commercial academic purposes while respecting intellectual property and fair-use principles.

The list prepared in 2014 served as the basis for the continued surveys in 2019 and 2023. In the 2019 survey, five journals changed their titles but were consistently tracked using unique identifiers (e.g., *Age* was renamed *Geroscience* in 2017^44^). In the 2023 survey, no journal changed its title.

**Fig. 1:**
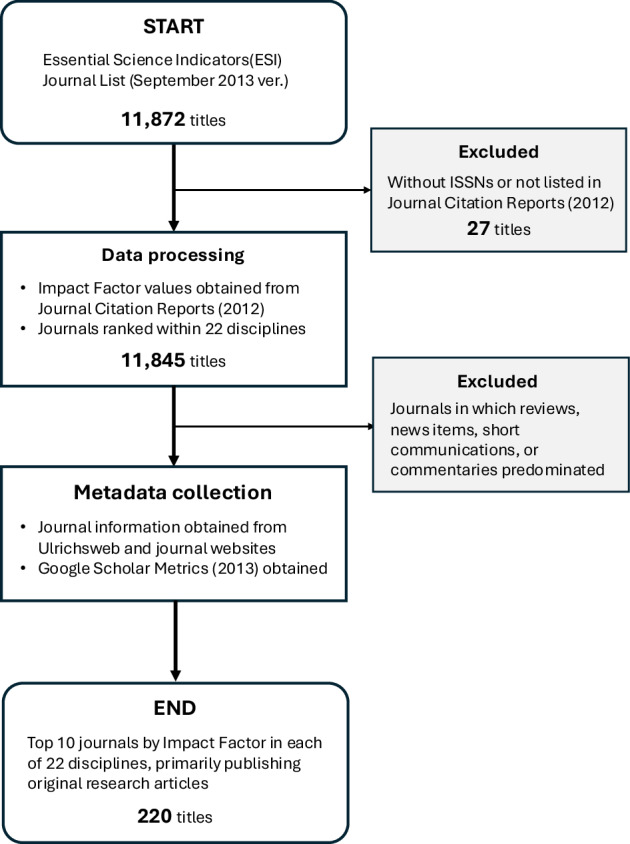
Flowchart of journal selection methodology for 2014 baseline survey.

A flowchart summarising the methodology of journal selection for the 2014 baseline survey is shown in Fig. 1.

### Policy review (2014, 2019, 2023)

Using the journal list, the submission guidelines and related documents of the target journals were reviewed to identify whether they included statements on data sharing. Two modes of data sharing were examined: deposition in repositories and inclusion as supplementary materials.

The most relevant statements were identified through a structured three-step search procedure:

1. **Locate author instructions:** The *Guidelines for Authors*, *Submission Instructions*, or similar sections were reviewed to identify documents describing journal policies.
2. **Inspect data-related subsections:** Within these pages, sections such as *Availability of Data*, *Data Distribution*, *Preparing Additional Files*, or *Sharing Datasets* were examined. The corresponding headings were recorded in the columns *DSP_title* and *SMP_title* for reference in subsequent surveys.
3. **Conduct site-specific search:** If no relevant statements were found, a site-specific Google search was performed using the query term “data” to locate all mentions of data sharing across the journal’s website.

This systematic approach ensured that diverse policy statements, including those embedded in subpages, were consistently identified and documented.

When a statement on data sharing was found, the strength of the requirement was classified into four categories, according to the criteria listed in Table 1. The classification codes were recorded in the dataset.

**Table 1:** Classification criteria for data sharing policy strength.

Code	Type	Phrase
3	require	require, request, mandatory, must, should, condition of publication, please deposit…
2	recommend	encourage, recommend, expect, support, if possible…
1	accept	accept, allow, might, possible to include, can be published…
0	no policy	(no mention)

Data sharing policies sometimes consist of journal-specific policies and, in other cases, publisher-level policies. However, these distinctions were not made in the dataset.

In some instances, data sharing requirements apply only to particular types of data, or the strength of the requirements varies by data type. In such cases, the most applicable category was recorded.

Some policies require the authors to include links to publicly available data. When it was unclear whether such statements referred to the data generated and shared by the authors themselves, the policy was classified as *no policy*.

For supplementary materials, data are sometimes made available through the journal platform, even in the absence of an explicit policy. In such cases, the policy was coded as *no policy*.

In the 2014 survey, designated or recommended repositories were recorded to capture disciplinary patterns and identify journals that guided authors in data deposition. From 2019 onward, the focus shifted to data citation policies to evaluate the adoption of citation-based incentives for data sharing following the release of the FORCE11 Joint Declaration of Data Citation Principles (JDDCP)^30^ in 2014.

### Survey periods

The data were collected over the following periods:

- 8 April to 8 May 2014
- 15 March to 26 May 2019
- 1 to 29 May 2023

### URL archiving and persistence check

After completing the policy review, archived versions of the reviewed webpages were retrieved using the Internet Archive (Wayback Machine)^45^ API to ensure the persistence of policy URLs. For each record, the script first attempted to locate the Data Sharing Policy (DSP) page. If unavailable, the Supplementary Materials Policy (SMP) page was used, and if neither was available, the journal homepage snapshot was recorded instead. Whenever possible, the snapshot closest to each survey point (2014, 2019, and 2023) was selected.

Access and reuse of these archived webpages comply with the Internet Archive Terms of Use^46^, which permit non-commercial academic use of publicly available materials while respecting the rights of original content owners.

## Data Records

The dataset^47^ described in this Data Descriptor is publicly available on *figshare* under a CC BY 4.0 licence at 10.6084/m9.figshare.29984809. It consists of three main components: (i) journal data sharing policies (2014, 2019, 2023), (ii) journal metadata, and (iii) accompanying documentation (README).

Journal data sharing policies

- journal-data-sharing-policies-2014.xlsx
- journal-data-sharing-policies-2019.xlsx
- journal-data-sharing-policies-2023.xlsx

Journal metadata

- journal-metadata.xlsx
  Documentation
- README.md

Table 2 provides an overview of the structure and content of the data sharing policy files. In addition to the core review, supplementary information was gathered: in 2014, the names of designated or recommended repositories, and in 2019 and 2023, details of the data citation policies, including whether journals explicitly referred to the FORCE11 Joint Declaration of Data Citation Principles (JDDCP)^30^. For full details, see the individual files.

**Table 2:** Data structure and content of data sharing policy files.

Header	Description
No	Unique identifier
ESI_category	Essential Science Indicators’ category (subject area)
TITLE	Journal title
PUBLISHER	Publisher’s name
ISSN	ISSN
IF	Impact Factor
URL	Journal URL
DSP_type	Data sharing policy type
DSP_title	Data sharing policy title
DSP_phrase	Key phrase used to determine type
DSP_description	Full text of data sharing policy
DSP_url	Data sharing policy URL
DSP_archive	Archived URL
SMP_type	Supplementary materials policy type
SMP_title	Supplementary materials policy title
SMP_phrase	Key phrase used to determine type
SMP_description	Full text of supplementary materials policy
SMP_url	Supplementary materials policy URL

Table 3 summarises the structure and content of the journal metadata file.

**Table 3:** Data structure and content of journal metadata.

Header	Description	Source
No	Unique identifier	
ESI_category	Essential Science Indicators’ category (subject area)	ESI Journal list (Sept 2013 ver.)
TITLE	Journal title	Journal Citation Reports/Journal website
PUBLISHER	Publisher’s name	Ulrichsweb
COUNTRY	Publisher’s country	Ulrichsweb
YEAR	Publication start year	Ulrichsweb/Journal website
ISSN	ISSN	Journal Citation Reports/Journal website
URL	Journal URL	Journal website
IF	Impact Factor	Journal Citation Reports
GSM h5-index	GSM h5-index	Google Scholar Metrics

Publisher names were standardised by consolidating regional subsidiaries (e.g., Springer New York LLC and Springer France into Springer) and grouping publishers within the same corporate entity (e.g., Elsevier BV and Pergamon into Elsevier). Five journals changed titles between survey periods but were tracked consistently using unique identifiers.

**Fig. 2:**
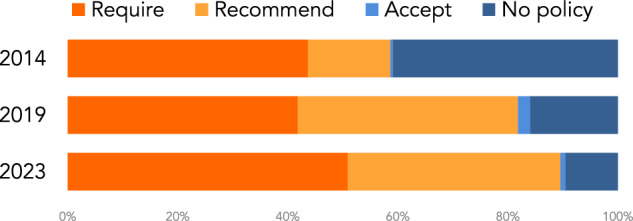
Distribution of the data sharing policy requirements across 220 journals for 2014 2019 and 2023.

**Fig. 3:**
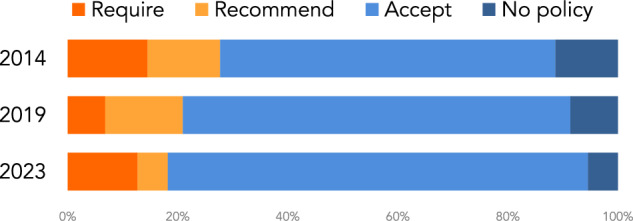
Distribution of the supplementary materials policy requirements across 220 journals for 2014 2019 and 2023.

The distribution of the data sharing policy requirements across the 220 journals for 2014 2019 and 2023 is shown in Fig. 2. The distribution of the supplementary materials policy requirements across the same set of journals is shown in Fig. 3.

**Fig. 4:**
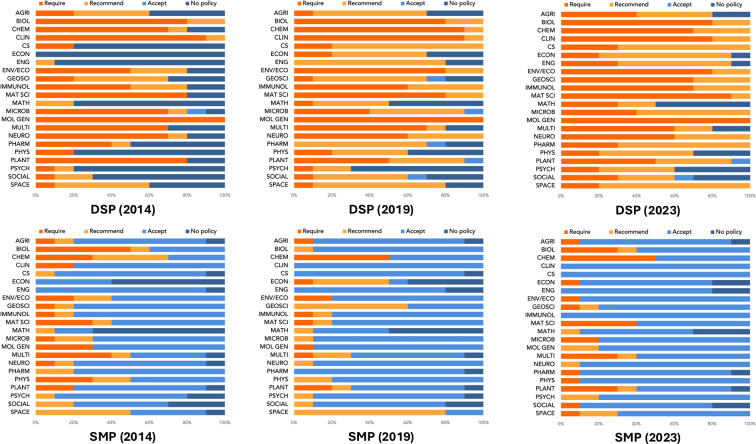
Disciplinary distribution of data sharing and supplementary materials policy requirements across 220 journals for 2014 2019 and 2023. *Note*. Abbreviations were defined by the author based on the 22 subject categories of the Essential Science Indicators Journal List (Clarivate). Full field names are available at https://esi.help.clarivate.com/Content/journal-list.htm.

Fig. 4 presents the distribution of data sharing and supplementary materials policy requirements by discipline for 2014 2019 and 2023. This figure provides a field-level perspective, revealing variation across disciplines and an overall strengthening of data sharing requirements over time.

## Technical Validation

### Data collection and coding process

The data from 2014 were collected and coded solely by the author. For the 2019 and 2023 surveys, ten trained research contributors (nine for 2019 and one for 2023) conducted an initial review and classification of policies following a shared coding protocol. The author subsequently verified and corrected all entries to ensure cross-year consistency and to minimise inter-rater variation.

### Quality assurance procedures

Prior to data publication, all data sharing policies were re-examined to improve classification consistency across the three survey periods. The classification criteria (Table 1) were refined based on accumulated experience from the three surveys to enhance the reliability of the assessments of policy strength.

### Classification refinement and validation

Key refinements involved the interpretation of policies requiring links to publicly available data. In earlier classifications, such requirements were often coded as *require* under data sharing policies. However, such language does not always clearly refer to data generated and shared by the authors themselves and could instead refer to existing public datasets or databases. To maintain consistency with the focus on author-generated data sharing, these ambiguous cases were reclassified as *no policy* when the policy wording was not explicit.

To enhance transparency and reduce potential subjectivity, an additional validation step was conducted using a large language model (ChatGPT-5o, OpenAI, 2025). The model was provided with the classification criteria in Table 1 and the full text of each policy description and was prompted as follows (translated from the Japanese instruction used in the verification process):

*“Read the policy description below and, using the classification criteria (‘require’ = 3, ‘recommend’ = 2, ‘accept’ = 1, ‘no policy’ = 0), determine the most appropriate category based on the phrases included.”*

The model’s suggestions were compared with the existing codes, and all discrepancies were manually reviewed by the author.

As a result of this comprehensive review process, the classification codes were revised for 22 data sharing policy entries and four supplementary materials policy entries across the three survey periods. Specifically, the data sharing policy classifications were updated for six journals in 2014, six journals in 2019, and ten journals in 2023. The supplementary materials policy classifications were updated for one journal in 2014, one journal in 2019, and two journals in 2023. These revisions improved the overall consistency and accuracy of policy strength assessments, with particular attention paid to distinguishing policies that explicitly required author data sharing from those with broader requirements for data availability.

### Inter-rater reliability assessment

For the 2019 and 2023 datasets, inter-rater reliability was assessed by comparing the initial classifications made by the research contributors with the final classifications established after author review.

### Data completeness and accuracy checks

All 220 journals were successfully surveyed across the three time points. Journal metadata (e.g., Impact Factor, publisher) were cross-verified using multiple sources, including Journal Citation Reports and publisher websites. Because some policy URLs may have become inactive since the surveys were conducted, corresponding archived URLs were additionally retrieved through the Internet Archive (Wayback Machine) API to ensure long-term accessibility. These archived URLs have been incorporated into the dataset to enhance traceability.

### Handling of journal title changes

Titles of five journals changed between 2014 and 2019. These journals were consistently tracked across all survey periods by maintaining their original identification numbers and recording both the old and new titles, thereby ensuring the longitudinal integrity of the dataset.

## Usage Notes

Descriptions of data sharing policies and supplementary materials policies may contain line breaks within individual spreadsheet cells.

The URLs correspond to those recorded at the time of data collection, and some may no longer be active. For transparency and long-term accessibility, archived versions of these webpages have also been included in the dataset via the Internet Archive (Wayback Machine)^45^. When an original link is inactive, users can refer to the corresponding archived URL provided in the dataset.

Users may also combine this dataset with external open access (OA) information resources, such as DOAJ (https://doaj.org/), to examine potential relationships between journal data sharing policies and OA status.

## Data Availability

The dataset supporting this Data Descriptor is publicly available on *figshare* under a CC BY 4.0 licence at 10.6084/m9.figshare.29984809.

## Code availability

No custom code was used in the generation or processing of the dataset.
